# 3D-Printed Bolus-Assisted Radiotherapy for Converting Unresectable Breast Cancer with a Breast Prosthesis into a Resectable Condition: A Case Report

**DOI:** 10.3390/curroncol33060335

**Published:** 2026-06-05

**Authors:** Shih-Kai Hung, Wei-Ta Tsai, Chun-Hung Lin, Moon-Sing Lee, Hon-Yi Lin, Liang-Cheng Chen, Chia-Hui Chew, Feng-Chun Hsu, Wen-Yen Chiou

**Affiliations:** 1 Department of Radiation Oncology, Dalin Tzu Chi Hospital, Buddhist Tzu Chi Medical Foundation, Chiayi 62247, Taiwan; 2 School of Medicine, Tzu Chi University, Hualien 97004, Taiwan; 3 Shin-Ho Biotech Co., Ltd., Miaoli County 366003, Taiwan; 4 Department of Biomedical Imaging and Radiological Sciences, National Yang Ming Chiao Tung University, Taipei 112304, Taiwan; 5 Department of General Surgery, Dalin Tzu Chi Hospital, Buddhist Tzu Chi Medical Foundation, Chiayi 62247, Taiwan

**Keywords:** 3D-printed, bolus, radiotherapy, breast cancer

## Abstract

**Background**: The use of conventional boluses in recurrent breast cancer often fails to conform to irregular surfaces, leading to air gaps and suboptimal dose distribution. We present a clinical experience involving a 3D-printed conformal bolus for a patient with gross recurrence and breast prosthesis invasion—a complex scenario where the treatment goal was surgical conversion. This report aims to generate hypotheses regarding the utility of customized boluses in facilitating the resection of initially unresectable tumors in the presence of reconstructive hardware. **Case Presentation**: A 58-year-old female with a history of breast cancer and prosthesis reconstruction presented with a rapid chest wall recurrence in 2018. The tumor invaded the overlying skin and the underlying prosthesis, rendering it unresectable. The patient received intensive salvage radiotherapy using Volumetric Modulated Arc Therapy (VMAT) with a dose-escalation regimen and a customized 1 cm 3D-printed bolus. While daily IGRT/CBCT and in vivo dosimetry were not available to definitively quantify the air gap reduction, the technical application of the bolus aimed to optimize surface dose coverage. Two months post-treatment, significant tumor regression was observed, allowing for the successful surgical removal of both the tumor and the prosthesis. **Conclusions**: To our knowledge, this case illustrates a specialized application of 3D-printed boluses in complex salvage scenarios. Following treatment, the patient experienced improved quality of life through pain reduction and reduced dressing frequency. Although the dramatic tumor response likely reflects the overall intensity of the radiotherapy regimen, our experience suggests that a 3D-printed bolus is a physically plausible tool to aid in achieving local control and facilitating surgical intervention. Further prospective studies are required to isolate the specific dosimetric advantages of this technology over conventional methods.

## Simple Summary

Treating recurrent breast cancer on an irregular chest wall, especially when a breast implant is involved, is a major clinical challenge. Conventional skin covers used during radiation therapy often leave air gaps, leading to imprecise radiation delivery. This case report describes a 58-year-old female patient with an aggressive recurrence that invaded both her skin and breast implant, making surgery initially impossible. By using a customized, 3D-printed skin cover, the medical team optimized the radiation dose delivered to the uneven tumor surface. Following this intensive treatment, the tumor shrank significantly, allowing for the successful surgical removal of both the tumor and the implant. This experience suggests that 3D-printing technology can be a highly effective tool to help doctors deliver precise radiation therapy, achieve better tumor control, and successfully convert previously inoperable cases into candidates for surgery.

## 1. Background

Large field tumor recurrence over the skin is usually difficult to treat clinically. Megavoltage radiotherapy is one of the salvage treatment options for local recurrent breast cancer, especially for unresectable lesions. However, the megavoltage photon beam X-ray that has been adopted to treat malignant tumors has a skin-sparing effect, which could reduce skin radiation dose and skin side effects [1]. Because of the skin-sparing effect, the shallow skin surface area may receive less radiation dose than deeper regions, and to avoid this limitation, a bolus is often used with megavoltage radiotherapy.

The bolus has been used in radiotherapy with various materials for over half a century. A human tissue-simulated material or near tissue-equivalent material has been used as a conventional bolus of different thicknesses. The limitation of the conventional bolus is that it does not conform to the patient’s body surface and may result in air gaps. According to the research of Martin J. Butson et al. [2], an air gap of 4 mm will introduce an approximately 0–4% reduction in surface dose and up to 10% dose reduction with a 10 mm air gap. Boman et al. also found that for Volumetric Modulated Arc Therapy techniques, the presence of 10 mm air gaps reduced the surface dose on average by 13.6% according to their Monte Carlo investigation [3]. Furthermore, the conventional bolus is a flat soft material which does not fit to the body shape well, leading to an inaccurate bolus position during the radiotherapy course [4].

A patient-specific three-dimensional printed (3D-printed) bolus has been used in clinical practice in recent years [5]. A 3D-printed bolus is produced according to the patient’s real body shape, which may reduce the skin-sparing effect and improve placement location accuracy compared with the conventional bolus, thereby increasing the tumor surface radiation dose. Accordingly, a 3D-printed bolus could potentially improve local tumor control. As was concluded by Park, Choi et al. [4] “Customized 3D-printed bolus has been shown to potentially replace and improve upon commercially available boluses, potentially reduce bolus placement errors and overcome some of the disadvantages in traditionally made boluses”.

In some specific body areas, such as the nose, ear, and scalp, the 3D-printed bolus has frequently replaced the conventional bolus to fit the body’s curvature [5]. Many clinical teams have tested different materials for the 3D-printed bolus to evaluate the dose distribution characteristics. Polylactic acid is a commonly used 3D-printed material for tissue equilibrium [6,7].

In the setting of salvage radiotherapy for locally advanced breast cancer, managing a huge fungating tumor with a highly uneven and irregular surface presents a formidable dosimetric challenge, particularly when compounded by the presence of an underlying breast prosthesis. Conventional commercial boluses often fail to provide adequate anatomical conformity in such complex topographies, leading to significant air gaps. According to recent evidence, while dose attenuation remains clinically manageable for minor gaps, air gaps exceeding 2 cm result in pronounced dose reduction in the superficial skin zone and target volumes, potentially compromising local control [8]. To bridge the gap toward surgical resection in otherwise inoperable cases, achieving precise dose escalation is paramount. Therefore, utilizing a 3D-printed bolus is essential in this scenario to eliminate excessive air gaps, ensure optimal surface dose distribution, and provide the precise target coverage necessary for effective cytoreduction and subsequent surgical salvage.

Moreover, while 3D-printed boluses have been increasingly documented in the recent literature, this case is unique for its specific clinical challenge: a gross recurrence with prosthesis invasion where surgical conversion was the primary treatment goal. To our knowledge, this is one of the few reports to detail the successful transition from an initially unresectable recurrent breast tumor and fixed prosthesis to a resectable state following radiotherapy optimized with a 3D-printed bolus. The novelty of this work lies not in the bolus technology itself, but in demonstrating its utility as a critical component in a salvage strategy aimed at facilitating surgical intervention in patients with complex, underlying reconstructive hardware.

## 2. Case Presentation

A 58-year-old female was diagnosed with invasive carcinoma of the left breast in November of 2016. The patient received left modified radical mastectomy with pathological stage IIB, pT2N1aM0, and simultaneous breast reconstruction with a breast prosthesis. The pathology of the first definite surgery showed invasive carcinoma, grade III, with lymphovascular permeation, with two axillary lymph node-positive, ER(+), PR(−), Her-2(3+) tumors.

The patient subsequently received a complete course of adjuvant chemotherapy with doxorubicin and cyclophosphamide, then Taxotere and Herceptin, and underwent their first radiotherapy course in October 2017 with total 5040 cGy to left whole breast, axilla, and SCF, with a daily dose 180 cGy, which was then followed by tumor bed boost 1000 cGy.

In May of 2018, local regional recurrence over the left chest wall and right axillary lymph nodes was suspected. Simultaneous wide excision of the recurrent left chest wall tumor and right axillary lymph node dissection were performed. The pathology of the second salvage surgery showed recurrent invasive carcinoma of no special type, grade III, with multiple lymphatic invasion, ER(−), PR(−), Her-2(2+), with deep resection margin involvement with right axillary lymph nodes metastases (10/13) with extranodal extension, rpT4aN3aM0, rp-stage IIIC.

**Figure 1:**
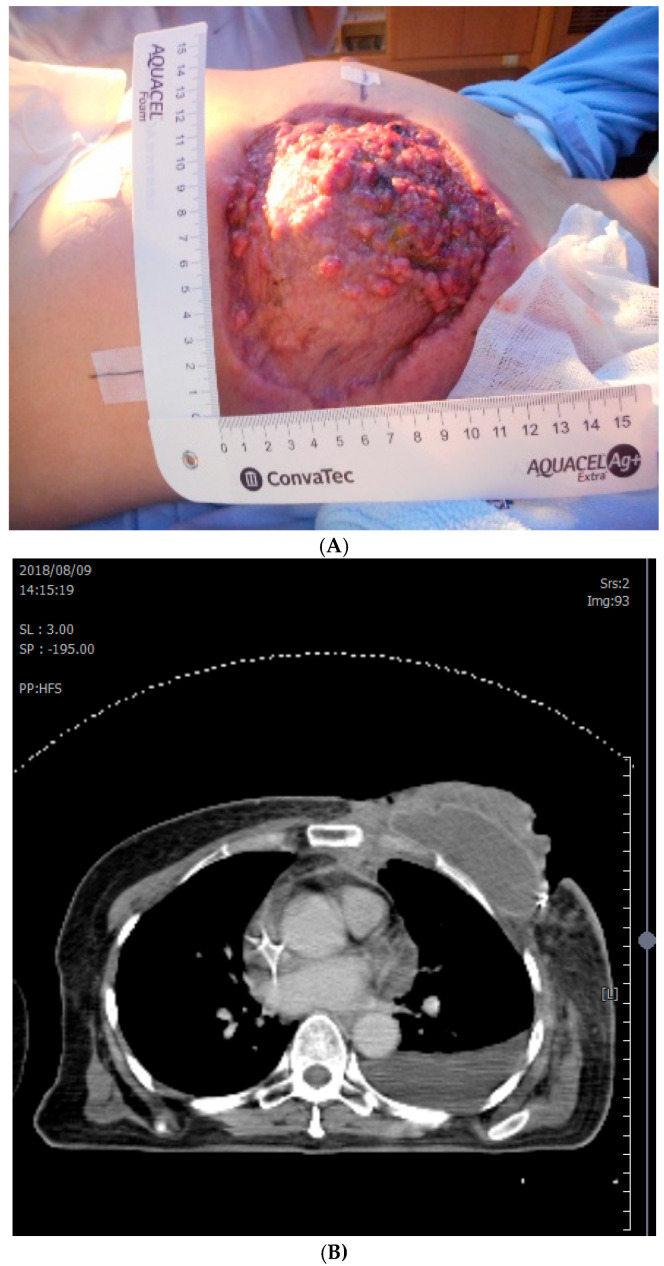
Clinical and radiological presentation of the aggressive chest wall recurrence. (**A**) Clinical image of the left chest wall tumor invading the whole skin above the left breast prosthesis, in August of 2018, just 2 months after second salvage surgery in June 2018. (**B**) CT image of the thick large tumor invading the whole skin above the left breast prosthesis and the nearby soft tissue close to the sternum, making the tumor unresectable and the prosthesis unremovable.

However, in July of 2018, just 2 months after the second salvage surgery in May 2018, local recurrence over the same left chest wall was noted and the recurrent tumor grew very rapidly and invaded the whole skin above the left breast prosthesis and invaded the nearby surrounding soft tissue close to the sternum, making the tumor unresectable and the prosthesis not removable (Figure 1A,B). Clinical staging was rrcT4bN0M0, grade III, ER(−), PR(−), rrc-stage IIIC, with the T4b designation attributed to ulceration and ipsilateral skin skip macroscopic metastases on the left chest wall. Notably, there was no pathological evidence of pleural effusion or other distant metastases.

Because of the severe pain caused by the large fungating tumor wound needing frequent wound dressing changes, a second salvage radiotherapy was indicated. Chemotherapy was not considered at that time as the tumor enlarged very rapidly, and due to the previous poor response to chemotherapy. A third surgery is also not possible due to the tumor rapidly invading the prosthesis peripheral soft tissue, which was confirmed after consulting a surgeon.

Given the local advanced nature of disease status at that time, the clinical strategy involved dose-escalated radiotherapy. The objective was to maximize loco-regional control and prevent further cutaneous recurrence while maintaining a potentially curative intent.

Due to the unresectable, rapidly enlarging recurrent breast tumor and poor response to prior chemotherapy and targeted therapy, local salvage re-radiotherapy was the only remaining clinical option. Reflecting the aggressive nature of the recurrence, the interval between the completion of the initial radiotherapy and the initiation of the second course was only 7 months (from December 2017 to July 2018). Notably, within just two months following salvage surgery with clear margins, the tumor progressed rapidly into multiple visible tumor nodules (Figure 1A) that completely encapsulated the breast implant and invaded medially into the sternum and intercostal muscles (Figure 1B), rendering them unresectable. Urgent intervention was imperative to arrest this rapid progression and prevent catastrophic local complications; hence, a salvage dose of 62.5 Gy in 25 fractions was prescribed. Although the cumulative radiation dose was substantial, the treatment plan was optimized using Volumetric Modulated Arc Therapy (VMAT) to ensure patient safety. Under this advanced planning technique, the critical organ-at-risk (OAR) doses for the second course were maintained at acceptable levels, with a heart Dmean of 16.28 Gy and an ipsilateral lung V20 of 28.6% (compared to a heart Dmean of 9.78 Gy and left ipsilateral lung V20 of 12.0% in the first course). The resulting dose distribution was deemed clinically acceptable given the clinical urgency and the necessity of aggressive local control.

**Figure 2:**
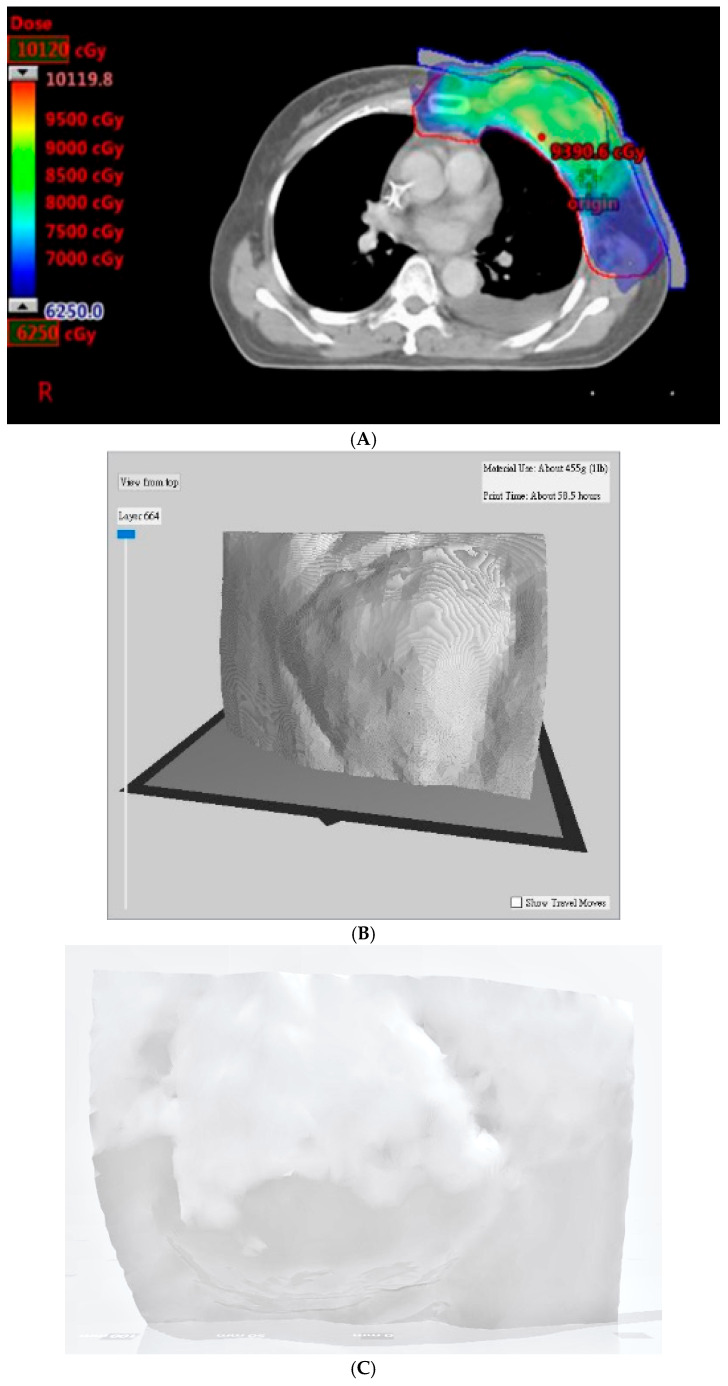

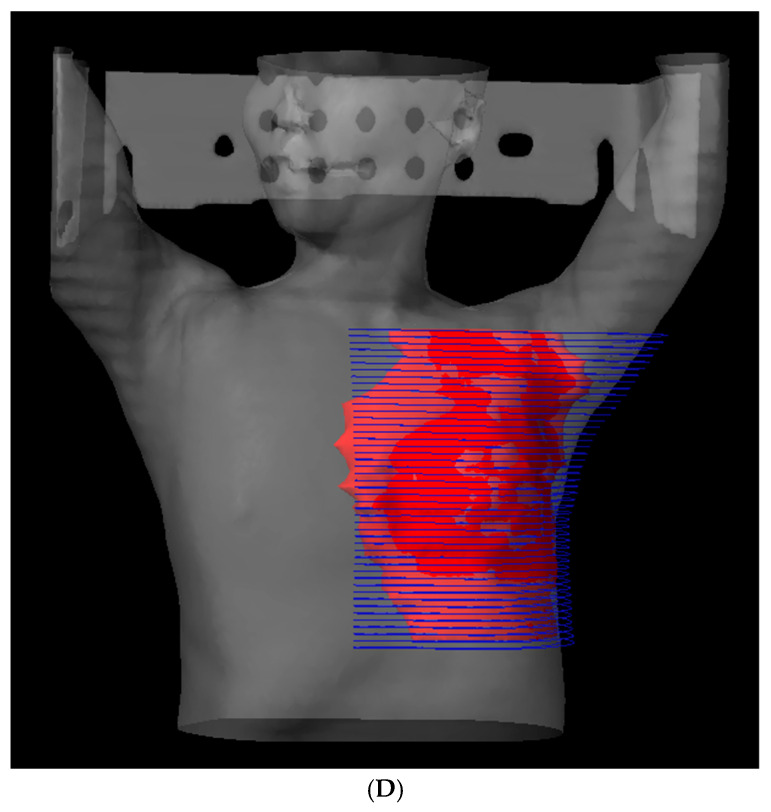
Workflow of the 3D-printed bolus design and treatment planning. (**A**) The Eclipse treatment planning system from Varian medical systems, with a virtual bolus, for the calculation of dose distributions. (**B**) The body surface curvature data and bolus shape parameter were exported from the TPS to a 3D-printed software. (**C**) The customized 3D-printed bolus for this patient. (**D**) The GTV (red area) and the bolus placement site (blue fence line) of salvage radiotherapy course.

To cover both the deeper tumor region and the region of surface skin invasion, a treatment design using a 6 megavoltage photon beam with a bolus was adopted. The analytical anisotropic algorithm was implemented in the Eclipse (Varian Medical Systems, Palo Alto, CA, USA) treatment planning system (TPS), version 13.6, for the calculation of dose distributions. The Volumetric Modulated Arc Therapy technique was used on a Varian TrueBeam (Varian Medical Systems, Palo Alto, CA, USA) linear accelerator. Because of the irregular shape of the chest wall gross tumor, as shown in Figure 1A, we customized a 3D-printed 1 cm bolus to cover the patient’s left chest wall during radiation therapy and provide an adequate skin dose. After importing the patient’s simulation images (Figure 1B), the TPS was used to add on a virtual bolus and calculate the suitable bolus thickness (Figure 2A). Then, the body surface curvature data and bolus shape parameter were exported from the TPS to a 3D-printed software (version 1) (Figure 2B) [9]. This 3D-printed bolus was produced by the Flashforge 3D printer (Zhejiang Flashforge 3D Technology Co., Ltd., Hangzhou, China) with the polylactic acid (PLA) material.

The patient-specific bolus was designed based on the planning CT images and manufactured using a Flashforge 3D printer (Zhejiang Flashforge 3D Technology Co., Ltd.) with PLA filament. The clinical application of this 3D-printed accessory was approved by the Institutional Review Board of Dalin Tzu Chi Hospital (IRB No: B10903005). Before clinical implementation, the bolus was subjected to a standardized quality assurance (QA) process, which included a CT scan to verify its internal infill density and ensure its water-equivalent thickness was consistent with the dose calculation in the treatment planning system.

This 3D-printed bolus can fit with the patient’s body surface without producing large air gaps and is stable for daily treatment positioning (Figure 2C). The second salvage radiotherapy field covered the left whole breast (covering the left chest wall skin skip metastatic lesions, including GTV with 0.5 cm margin to form CTV), as shown by the red area (Figure 2D). The main overlapping field of the two courses of radiotherapy is the left whole breast, including the entire breast prosthesis underlying the gross tumor. Initial and salvage radiotherapy plans were compared. For the first course, doses to OARs were as follows: heart Dmean 978.2 cGy, left lung V20 12.0%, right lung V20 0%, and spinal cord Dmax 906.1 cGy. In the salvage course, these values were 1628.3 cGy, 28.6%, 8.9%, and 1546.6 cGy, respectively. No interruption occurred during the salvage radiotherapy course.

Initially, the patient required dressing changes four times daily for this large fungating tumor, antibiotic therapy, and morphine for pain management.

**Figure 3:**
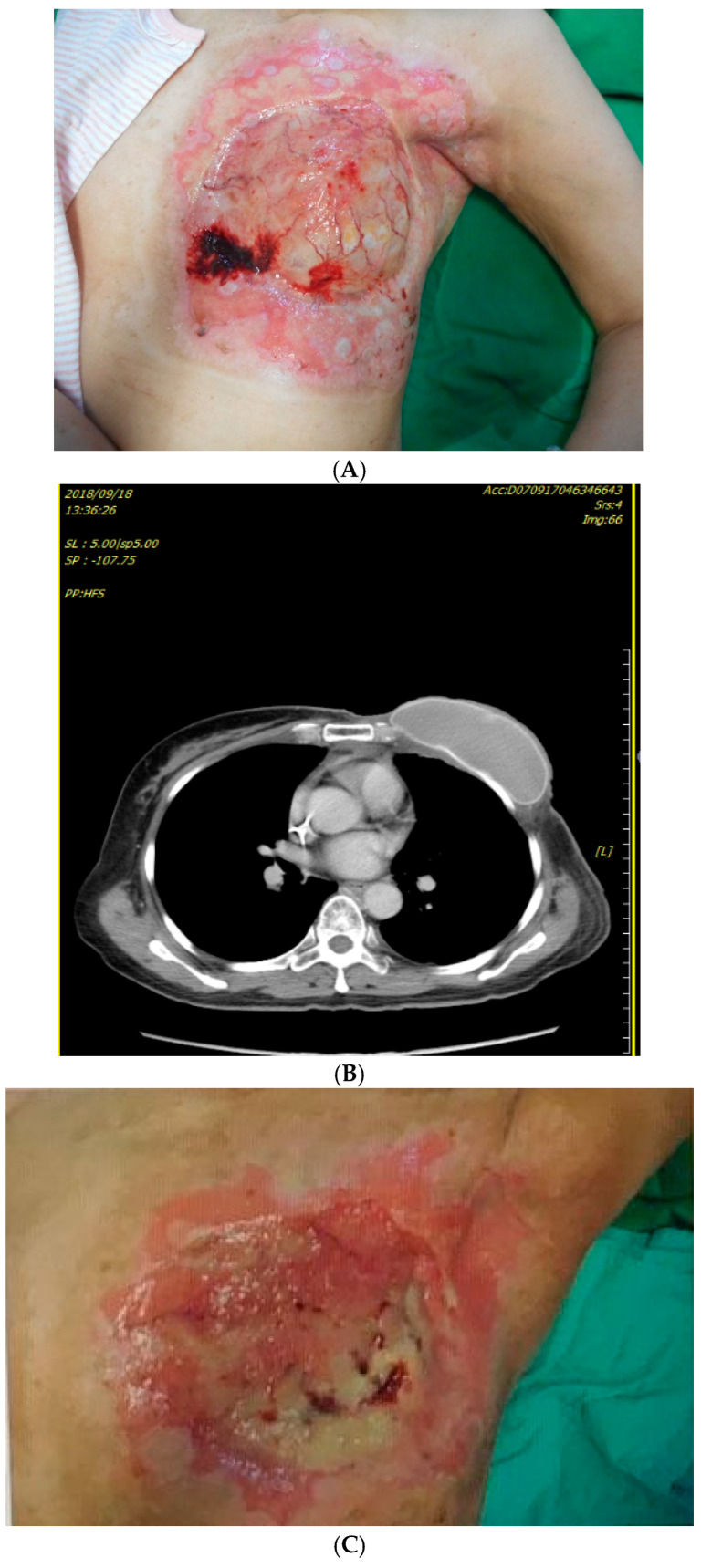
Treatment response, skin toxicities, and final surgical intervention. (**A**) Grade III skin reaction was observed during treatment. (**B**) Two months after salvage radiotherapy, in September 2018, obvious skin carcinomatosis tumor regression was noted. (**C**) The breast prosthesis was removed and the local residual microscopic tumor was resected successively.

During treatment, grade I to III skin reactions (Figure 3A) were observed. In this case, close skin reaction observation and timely skin care intervention was crucial after the skin tumor regressed with skin defects. After the salvage radiotherapy treatment course was finished, the skin reaction ameliorated. Two months after salvage radiotherapy, in September 2018, obvious skin carcinomatosis tumor regression was noted (Figure 3B) compared with the previous thick tumor above the breast prosthesis (Figure 1B). Because this initial unresectable local recurrent tumor regressed effectively after radiotherapy, salvage surgery became possible.

Finally, the breast prosthesis was removed, and the local residual microscopic tumor was resected successively (Figure 3C) in October 2018. The pathology of the third salvage surgery showed chest wall soft tissue necrosis, with right axilla metastatic carcinoma (4/4) with extracapsular spreading, with microscopic satellite nodules, ER(−), PR(−), Her-2(2+). After the third surgery with the prosthesis removed, the regimen was simplified to daily dressing changes and Ultracet (Tramadol 37.5 mg and Acetaminophen 325 mg), with no further need for antibiotics or morphine.

However, only one month after the successful third salvage surgery, liver metastasis was detected in November 2018. Despite receiving further systemic treatments—including ado-trastuzumab emtansine (Kadcyla), gemcitabine (Gemzar), vinorelbine, lapatinib (Tykerb), capecitabine (Xeloda), and picibanil (OK-432)—the patient passed away in February 2019.

## 3. Discussion

Kwangwoo Park et al. [10] and Daniel F. Craft et al. [6] published a report on the use of the 3D-printed bolus in post-total mastectomy adjuvant electron conformal therapy in 2017. Unlike previous reports, the patient described in this study received a prosthesis implant during initial breast cancer surgery, which separated the surface skin metastasis and deeper muscle lesion. The rapid recurrent tumor above and around the breast prosthesis resulted in an unresectable tumor and prosthesis. The prosthesis divided the shallow thin skin and underlying muscle layer and made the chest wall skin tumors become a very superficial tumor, which made it unsuitable for conventional radiation therapy. In this complicated situation, radiotherapy must reach an adequate dosage for both the very superficial tumor and the peripheral deep tumor. With the 3D-printed technique, a customized bolus can decrease the air gap to the greatest extent.

Compared with those of the traditional flat bolus, the benefits of the 3D-printed bolus are that it reduces the air gap and enhances the bolus placement accuracy during the daily treatment course. Therefore, it reduces the difference between the TPS dose distribution and the real dose that is received by the patient. With accurate dose delivery, radiation oncologists can predict the effect on skin and introduce skin care interventions as early as possible.

Of the traditional bolus materials, manually manufactured wax is another conventional bolus option for the treatment of irregular surface regions [11]. Christine Albantow et al. compared the 3D-printed nose bolus to the traditional wax bolus for volumetric accuracy, dosimetric effect, and cost-effectiveness (bolus manufacture time and material costs) in 24 patients with nose tumors. The authors concluded that the 3D-printed bolus could replicate the virtual bolus geometry on radiotherapy TPS and was less costly to manufacture when compared with the wax bolus [12].

To address the challenges of the skin air gap, a 2022 study demonstrated that 3D-printed boluses significantly enhance the precision of post-mastectomy chest wall radiation therapy [13]. The research highlights that this technology achieves superior physical conformity, reducing the mean air gap to just 1.01 mm. This improved fit maintains high dosimetric accuracy—with an absolute percentage dose difference of approximately 1.77%—effectively mitigating the air gaps and dosage inconsistencies often encountered with traditional commercial boluses.

In addition to improving surface fit, 3D-printed boluses have demonstrated a superior ability to spare organs at risk compared to conventional methods. Recent comparative research showed that 3D-printed boluses reduced the mean dose to the heart and ipsilateral lung by an average of 0.8 Gy each [14]. This led to a significant reduction in normal tissue complication probability—decreasing by 0.14% for the heart and 0.45% for the lung (*p* < 0.05). These dosimetric gains were achieved alongside improved target conformity (conformity index of 0.83 vs. 0.80), suggesting that 3D-printing technology not only ensures target coverage but also plays a critical role in minimizing long-term cardiopulmonary exposure.

The pathology report from the third salvage surgery revealed “chest wall soft tissue necrosis” approximately two months after the completion of high-dose re-irradiation. While this may reflect treatment-related tissue injury, tumor necrosis as a component of the radiotherapy response cannot be excluded. Nevertheless, this finding did not compromise the subsequent surgical management. Because the recurrent tumors were localized to the skin and chest wall overlying the breast implant, necrosis of either the tumor or the normal tissue in this superficial area had no negative impact on the procedure. In fact, the shrinkage of tumors adjacent to the implant (e.g., near the sternum) rendered the surgical intervention feasible. This clinical outcome underscores the critical necessity of promptly initiating secondary salvage radiotherapy. Had the tumor bypassed the implant and infiltrated the underlying deep chest wall, any subsequent surgical options would have been rendered entirely impossible.

Delivering high-dose re-irradiation in the presence of a breast prosthesis significantly escalates the risk of implant-related complications. Radiation is well-documented to induce severe tissue fibrosis and capsular contracture around breast reconstructed fields, substantially increasing the rates of implant exposure, extrusion, or reconstructive failure [15]. Furthermore, managing radiotherapy in the setting of an open, ulcerated wound presents distinct clinical and dosimetric challenges. Irradiation of active malignant fungating wounds is often performed to achieve tumor regression, control symptoms, and facilitate oncological wound management; however, it simultaneously compromises the surrounding tissue microenvironment and hinders physiological tissue repair, which can contribute to delayed local tissue necrosis [16]. In this case, the decision to proceed with aggressive re-irradiation required careful counterbalancing of these combined risks against the urgent necessity of local tumor control to maintain surgical resectability.

Another critical factor contributing to the observed tumor regression is the intensive dose-escalation regimen employed in this case. The patient received 62.5 Gy in 25 fractions, with a simultaneous integrated boost (SIB) increasing the tumor inner dose to 73 Gy. Historically, a short inter-treatment interval between primary radiotherapy and recurrence (<24 months) is established as a highly aggressive feature associated with poor locoregional control, necessitating a potent therapeutic response [17]. In this clinical context, achieving a high biological effective dose through a hyperfractionated or dose-escalated approach is inherently potent and remains a primary driver of dramatic clinical response in refractory recurrent breast cancer [18,19]. Therefore, it is plausible that the successful outcome reflects the cumulative efficacy of the intensified radiotherapy itself, rather than being solely attributable to the 3D-printed bolus. While the customized bolus was intended to optimize surface dose and conformality, its specific therapeutic benefit remains intertwined with the overall impact of the high-dose SIB protocol. This alternative explanation further emphasizes the need for future comparative studies to isolate the incremental value of 3D-printed boluses in dose-escalated settings.

## 4. Limitation

Despite the positive clinical observations, this study has several limitations regarding the direct attribution of the outcome specifically to the 3D-printed bolus. First, while the proposed mechanism—improved surface dose and target coverage—is physically plausible, we did not provide direct quantitative evidence or comparative planning (e.g., comparing a plan with a conventional bolus versus no bolus) to isolate the bolus’s specific impact from the overall effect of the radiotherapy regimen. Furthermore, in this clinical case presentation, the patient did not receive Image-Guided Radiation Therapy techniques, and no Cone-Beam Computed Tomography images were available to verify the daily setup or the precise fit of the bolus. The absence of in vivo dosimetry or phantom validation also means the actual dose enhancement was not empirically measured. Consequently, further comparisons—specifically regarding the definite air gap data between a conventional bolus and 3D-printed bolus—are needed in future studies to confirm these findings. Until then, the observed clinical outcome should be interpreted as a plausible correlation rather than a directly demonstrated causal link.

In addition to these methodological constraints, certain dosimetric data regarding organs at risk warrant acknowledgment. Specifically, the exact radiation doses received by the brachial plexus during both the initial and subsequent radiotherapy courses remain unreported. Although the axilla and supraclavicular fossa were included in the target fields, full dosimetric reconstruction for the brachial plexus was unavailable due to the retrospective nature of this case and the limitation of extracting historical treatment planning systems data. Nonetheless, it is worth noting that the patient demonstrated no clinical signs or symptoms of brachial plexus neuropathy throughout the entire treatment and follow-up period.

## 5. Conclusions

In summary, this case highlights the application of a 3D-printed bolus in salvage radiotherapy for a breast cancer patient with a fungating tumor and an underlying prosthesis. While this report should be viewed as a hypothesis-generating technical presentation rather than definitive evidence of the bolus’s causal role in achieving resectability, the clinical outcome was encouraging. Despite the patient ultimately succumbing to the disease, the treatment facilitated surgical intervention, provided dramatic pain relief, and significantly improved quality of life. We acknowledge that the specific contribution of the bolus, relative to the overall radiotherapy effect, remains to be quantitatively demonstrated through comparative studies and air gap data. Nonetheless, this experience suggests that customized 3D-printed boluses offer a plausible technical solution for complex clinical scenarios involving underlying prostheses, warranting further prospective investigation.

## Data Availability

The original contributions presented in this study are included in the article. Further inquiries can be directed to the corresponding authors.

## Abbreviations

OARs	Organs at risk
PLA	polylactic acid
3D-printed	three-dimensional printed
TPS	treatment planning system
VMAT	Volumetric Modulated Arc Therapy
